# Assessing healing of periapical pathologies by platelet-rich fibrin: A clinical study

**DOI:** 10.6026/973206300213912

**Published:** 2025-10-31

**Authors:** Deepak Sharma, Preeti Bhadouria, Pinky Chaurasia, Surbhi Patel, Devanshi Vaghela, Hemal Patel

**Affiliations:** 1 Department of Conservative Dentistry and Endodontics, Bharati Vidyapeeth (Deemed to be) University Dental College and Hospital, Navi Mumbai, India; 2 Department of Oral Medicine and Radiology, Maharana Pratap college of Dentistry and Reasearch Centre, Gwalior, Madhya Pradesh, India; 3 Department of Pedodontic and Preventive Dentistry, Triveni Institute of Dental Science and Research Center, Bodri, Bilaspur, Chhattishgarh, India; 4 Consultant Endodontist, Ahmedabad, Gujarat, India; 5 Department of Oral and Maxillofacial Surgery, Karnavati School of Dentistry, Karnavati University, Gandhinagar, Gujarat, India; 6 Department of Public Health Dentistry, Faculty of Dental Science, Dharmsinh Desai University, Nadiad - 387003, Gujarat, India

**Keywords:** Platelet-rich fibrin, periapical lesion, endodontic microsurgery, bone regeneration, guided tissue regeneration

## Abstract

One of the on-going clinical challenges in endodontic microsurgery is the regeneration of massive periapical lesions. Hence, a Forty patients participated in this randomized controlled clinical experiment to see how well platelet-rich fibrin (PRF) worked as a scaffold for regeneration in comparison to the body's inherent ability to mend blood clots. The decrease in lesion volume, pain ratings, and the repair of soft tissues were evaluated at 6 and 12 months by CBCT. PRF demonstrated a much higher rate of bone regeneration (85.2% vs. 64.8%, p < 0.001) and reduced levels of post-operative discomfort on days 1 and 3. Thus, we show that PRF is an inexpensive and easy way to improve the predictability of recovery after periapical surgery.

## Background:

Pulpal necrosis is the most common cause of peripheral periodontitis, an inflammatory disease affecting the tissues around a tooth's crown [1]. Although root canal therapy without surgery is the most common and effective method, it may fail in certain situations because of iatrogenic mistakes, complicate canal architecture, or long-lasting infections either within or outside the canals [2]. The gold standard for preserving natural dentition in these circumstances is endodontic microsurgery, which includes apicoectomy and root-end filling [3]. A critical factor for the long-term success of periapical surgery is the complete regeneration of the bone within the surgically created defect. While smaller lesions often heal by natural bone regeneration, larger through-and-through defects may result in incomplete healing or fibrous scar tissue formation [4]. In order to circumvent this restriction, guided bone regeneration (GBR) techniques have been developed, which include the use of different barrier membranes and bone grafts to direct osteoprogenitor cells while preventing the ingrowth of soft tissue [5]. However, these materials can be associated with additional costs, technique sensitivity, and potential for host immune reactions or disease transmission. Recent years have seen a boom in interest in autologous platelet concentrates a novel bioactive surgical adjuvant with the ability to hasten postoperative recovery. A second-generation platelet concentrate called Platelet-Rich Fibrin (PRF) was produced by centrifuging whole blood without anticoagulants by Dohan *et al.* [6].

Platelets, white blood cells, and other growth factors, such as vascular endothelial growth factor (VEGF), transforming growth factor-beta (TGF-β), and platelet-derived growth factor (PDGF), are trapped in a densely packed fibrin matrix [7]. Whereas PRP releases all of its growth factors at once, PRF releases them in stages, with each stage taking 7-14 days to completely activate. The regeneration of both hard and soft tissues relies on this ongoing stimulation of angiogenesis, cell proliferation, and differentiation [8]. The application of PRF has shown promising results in various dental and oral surgical procedures, including sinus floor augmentation, periodontal intrabony defect regeneration, and extraction socket preservation [9]. Preliminary studies and case reports have suggested its potential benefit in endodontic microsurgery, but robust clinical evidence from well-designed randomized controlled trials remains limited [10]. Furthermore, many earlier studies relied on two-dimensional periapical radiographs for assessing healing, which can be subject to distortion and superimposition. A more quantitative view of the extent to which bones are healing may be obtained with the use of cone beam computed tomography (CBCT). The purpose of this research was to address this gap in our understanding. Therefore, it is of interest to objectively assess the healing of periapical illnesses by comparing the radiographic and clinical outcomes of endodontic microsurgery with and without the concomitant use of PRF.

## Materials and Methods:

## Study design and sample selection:

The research used a prospective, randomized, controlled clinical trial design with a single center and a parallel-group layout. The expected 20% difference in bone fill across groups, together with a 15% standard deviation, 80% power, and a 0.05 significance level (α), led to the calculation of a sample size of 40 patients.

## Inclusion and exclusion criteria:

Patients aged 18-60 years who were referred for periapical surgeries on a single-rooted anterior tooth (incisor or canine) were screened.

## Inclusion criteria were:

[1] Presence of a persistent periapical lesion of endodontic origin following adequate non-surgical root canal treatment;

[2] A periapical lesion with a maximum diameter of ≥ 5 mm as measured on a preoperative CBCT scan;

[3] Good systemic health.

## Exclusion criteria included:

[1] Systemic diseases that could compromise healing (e.g., uncontrolled diabetes mellitus, immunosuppression);

[2] History of smoking (>10 cigarettes/day);

[3] Pregnancy or lactation;

[4] Known bleeding disorders or use of anticoagulant therapy;

[5] Vertical root fractures or periodontal disease affecting the involved tooth.

## Randomization and allocation:

Following the completion of all necessary assessments, patients were randomly assigned to either the Test Group (PRF Group, n=20) or the Control Group (n=20) using a computer-generated seed sequence. After the surgery was given a numerical value, a surgical assistant opened the opaque, sealed packets containing the allocation. The radiographic analyst who took the CBCT volume measurements had no idea which patient group each individual belonged No blinding of the surgeon or patients was possible because of the delicate nature of the procedure.

## Surgical protocol:

All surgical procedures were performed by a single calibrated endodontist.

[1] PRF preparation (Test Group only): A sterile vacutainer tube was used to extract 10 ml of venous blood from the patient's antecubital vein without anticoagulant just before surgery. Straightaway, the tube was spun in a centrifuge at 2700 rpm for duration of 12 minutes. The PRF clot that formed was then gently squeezed between sterile gauze to create a membrane after being removed from the red blood cell basis.

[2] Surgical intervention: The procedure continued with a local anesthetic (2% lidocaine with 1:100,000 epinephrine) and an elevated full-thickness mucoperiosteal flap. After gaining access to the periapical lesion by an osteotomy, it was thoroughly enucleated and then sent for pathological examination. A surgical bur was used to remove the root apex by 3 mm in a direction perpendicular to the tooth's long axis. After creating a 3 mm deep hole at the root tip using ultrasonic tips, the hole was filled with bioceramic root restoration material (MTA).

[3] Defectm:

1) Control Group: The bony cavity was irrigated with sterile saline, and hemostasis was achieved, allowing the defect to fill with a natural blood clot.

2) PRF Group: The prepared PRF membrane was folded and placed into the bony cavity, filling the defect.

[4] Closure: The flap was repositioned and sutured with 5-0 non-resorbable sutures.

## Post-operative care and follow-up:

All patients were prescribed amoxicillin 500 mg (three times daily for 5 days) and ibuprofen 400 mg (as needed for pain). Post-operative instructions were provided. Sutures were removed after 7 days.

## Outcome assessment:

[1] Primary Outcome (Radiographic Healing): CBCT scans were taken pre-operatively (baseline), and at 6 and 12 months post-operatively using a standardized protocol. The volume of the periapical radiolucency (in mm^3^) was measured by a blinded radiologist using specialized software (ITK-SNAP). The percentage reduction in lesion volume at each follow-up was calculated as: [(Baseline Volume - Follow-up Volume) / Baseline Volume] x 100.

[2] Secondary Outcomes (Clinical Assessment):

1) Post-operative Pain: Patients rated their pain intensity on a 10-point Visual Analog Scale (VAS; 0 = no pain, 10 = worst imaginable pain) at 1, 3, and 7 days post-operatively.

2) Soft Tissue Healing: Evaluated at the 7-day follow-up based on the Healing Index of Landry, Turnbull, and Howley.

## Statistical analysis:

We used SPSS (Version 25.0, IBM Corp.) to analyze the data. We used the Shapiro-Wilk test to make sure the data were normal. Age, lesion volume, and VAS scores were some of the continuous variables that were compared between the two groups using an independent samples t-test. The gender and tooth type categorical variables were tested using the chi-square test. A p-value lower than 0.05 was considered statistically significant.

## Results:

Forty individuals were considered for enrollment and randomization. After two patients in the control group were lost to follow-up, there were 38 patients remaining at the conclusion of the 12-month evaluation, 18 from the control group and 20 from the PRF group. All of the surgical incisions have disappeared without a trace. Table 1 (see PDF) displays the demographic and clinical data from the beginning of the study. It was determined that the randomization was effective since neither the PRF group nor the control group differed from one another in terms of age, gender distribution, tooth type, or initial periapical lesion volume (p > 0.05). The PRF group experienced significantly less post-operative pain compared to the control group at all measured time points (Table 2 - see PDF). The mean VAS scores were significantly lower on day 1 (p < 0.001), day 3 (p < 0.001), and day 7 (p = 0.002). Soft tissue healing was excellent in both groups, with no significant difference observed at the 7-day follow-up. Table 3 (see PDF) shows that the PRF group had a clear and substantial advantage in the main outcome, radiographic recovery. The PRF group had a mean percentage decrease in lesion volume of 62.5% during the 6-month follow-up, whereas the control group achieved only 41.3% (p < 0.001). By the 12-month follow-up, the difference had become much worse, with the PRF group reducing lesion volume by 85.2% compared to 64.8% in the control group (p < 0.001).

## Discussion:

Endodontic microsurgery using PRF as an adjuvant greatly improves the repair of periapical bone abnormalities and lessens postoperative pain, according to this randomized controlled clinical research. The null hypothesis was therefore rejected. The PRF group exhibited a substantially greater reduction in lesion volume at both 6 and 12 months, as quantified by precise CBCT analysis, confirming its efficacy as a regenerative biomaterial. The superior bone regeneration observed in the PRF group can be attributed to the unique biological properties of this autologous concentrate. The dense fibrin matrix of PRF acts as an ideal three-dimensional scaffold, promoting cell migration and proliferation while physically stabilizing the blood clot [11]. More importantly, this matrix entraps high concentrations of platelets and leukocytes, which slowly release a cocktail of key growth factors (PDGF, TGF-β, IGF and VEGF) over an extended period [7, 12]. In addition to their well-documented roles as powerful mitogens and chemo attractants for mesenchymal stem cells and osteoblasts, these factors are also pivotal in facilitating angiogenesis, the process that is vital for delivering cells and nutrients to the site of repair [13]. Our results are in line with those of earlier studies that have shown improved bone repair with PRF in various clinical situations, including periodontal regeneration and cystic defect obliteration [10, 14 and 15]. A significant secondary finding of our study was the marked reduction in post-operative pain in the PRF group. The control group, which healed via a conventional blood clot, reported significantly higher VAS scores, especially in the first 72 hours. The analgesic and anti-inflammatory effect of PRF may be mediated by the leukocytes entrapped within the fibrin matrix. While leukocytes are involved in the inflammatory response, they also secrete anti-inflammatory cytokines like interleukin-4 and growth factors that can modulate inflammation and downregulate pro-inflammatory pathways, leading to less swelling and pain [16, 17]. This clinical benefit enhances patient comfort and satisfaction, adding another dimension to the advantages of using PRF. The methodology of this study has several strengths, including its prospective, randomized controlled design, which minimizes selection bias. The use of CBCT for volumetric analysis of the periapical lesion provides a more accurate and objective assessment of three-dimensional bone healing compared to traditional 2D periapical radiographs, which are prone to geometric distortion and a lack of buccolingual information [15]. The standardization of the surgical protocol by a single operator further enhances the internal validity of our results. However, there are a few caveats to this research. There was just a 12-month follow-up period, and the sample size was tiny as well. Although there was noticeable healing, it would be helpful to evaluate the integrity of the regenerated bone with a longer-term follow-up of 2 to 4 years. As an additional caveat, the results may not be applicable outside of the specific setting where the research was carried out. To support these findings, more multi-center trials with bigger cohorts are necessary. It would be helpful to determine PRF's relative effectiveness by comparing it to other GBR materials, such as collagen membranes or bone grafts.

## Conclusion:

We show that autologous platelet-rich fibrin, when used as a scaffold for regenerative purposes in surgical defects after endodontic microsurgery, results in radiographic bone healing that is both faster and more comprehensive than natural clot formation, within the limitations of the study. Additionally, the application of PRF is associated with a significant reduction in immediate post-operative pain.
